# Hæmostatic Treatment of Cholera, Hæmorrhage, Exhaustion, Etc.

**Published:** 1863-12

**Authors:** E. H. Janes


HÆMOSTATIC TREATMENT OF CHOLERA, HÆMORRHAGE, EXHAUSTION, ETC.—*By E. H. Janes,* M. D.—The writer, when in India, had unexpectedly a regiment prostrated with fever placed under his charge, and having but a small supply of quinine, he employed tourniquets to intercept the blood in the extremities, with a result so favorable as to induce him to bring the subject to the notice of the profession. There being about twenty-eight pounds of blood in the human body, and about two pounds in each of the four limbs, to enable us to relieve the congestion attendant upon intermittent disease, we have the control of at least a pound of blood in each limb by retarding it in the veins. This may be done with advantage in the premonitory symptoms of apoplexy, in severe cases of dyspnœa, in some organic diseases, and inflammations, where it is equivalent to the withdrawal of a certain quantity of blood from the general system. Another method of controlling the circulation is by stopping the arterial circulation in a limb. If a tourniquet is applied to the femoral artery, probably half the blood intended for the limb is prevented passing into it and makes its way back to the heart, circulating through a smaller circle, and in some diseases, proving a powerful tonic or stimulating effect upon the general system. This is apparent in those sudden and appalling cases of uterine hæmorrhage, also in the collapsed stage of cholera, where the system is so much prostrated that the most powerful medicines have no effect, the application of the tourniquet immediately removes the painful cramps; it increases the volume of blood, which stimulates the heart to increased action, removes congestion, changes the morbid dis-distribution of blood from the secreting surface of the alimentary canal, and sets up a new and salutary action in its place. The purging and vomiting are thus stopped, the pulse becomes stronger, the heat and strength of the system are quickly restored, and time is allowed for medicines to act. When the individual is weak, and the state of collapse great, care is required in emptying by friction the blood in the veins of the extremity to be bandaged; and the effect will be more marked if the tourniquet be applied to four extremities. It may be kept on for hours, or even for a day or two. When reaction has taken place, the pressure of the tourniquet is complained of, and if it be loosened too abruptly the blood spreads over the extremities, and the patient rapidly sinks. A number of cases of cholera are reported, illustrating the treatment, and also the danger of a too sudden removal of the instrument, from which the following conclusions are deduced:—

1. By its obstructing the circulation it immediately stops the distressing cramps of the extremities in cholera.

2. By increasing the quantity of the circulating fluid in the trunk, and thereby stimulating the heart's action, it removes marbid congestions, stops the secretions from the bowels, increases the animal heat, and powerfully tends to restore health.

3. By improving the vigor of the system medicines act more powerfully, and in a more salutary manner, in removing morbid actions.

4. When the reaction has taken place, by loosening the tourniquets with care the determination of blood to the internal parts is diminished by its diffusion over the extremities upon which the tourniquet had been placed. They are immediately to be tightened when there is any coldness or weakness experienced, or any tendency to relapse. This must be most carefully watched for, and prevented.

5. By increasing the volume of blood in the contracted circulation, the force of the heart is increased, local congestions are removed, and the whole system is strengthened.

